# Analysis of notified drug poisoning among children in Santa Catarina state, 2016–2020

**DOI:** 10.1590/1984-0462/2024/42/2022155

**Published:** 2023-07-10

**Authors:** Karoliny Brock, Gabriel Oscar Cremona Parma, Alessandra de Sá Soares, Fabiana Schuelter-Trevisol

**Affiliations:** a Universidade do Sul de Santa Catarina, Tubarão, SC, Brazil.

**Keywords:** Poisoning, Child, Accidents, Intoxicação, Criança, Acidentes

## Abstract

**Objective:**

The aim of this study was to analyze the incidence of drug poisoning in children registered in the Santa Catarina Information and Toxicological Assistance Center between 2016 and 2020.

**Methods:**

This observational epidemiological study, with a historical cohort design, was carried out from reported cases of drug poisoning in children aged 0–12 years. Census sampling was used to collect data.

**Results:**

There were 4839 reported cases of drug poisoning among children in the State of Santa Catarina in the surveyed period, with an average annual incidence rate of 6 cases/1000 live births. The median age was 3 years. Most cases of poisoning occurred among girls aged 0–3 years by accidental ingestion of drugs at home. There was a predominance of signs and symptoms affecting the nervous system; only a small portion required hospitalization. Most cases were considered mild poisoning with a favorable outcome. No deaths were recorded. There was a tendency of increasing cases over time, however not significant. There is a predominance of incident cases in the Great West of the state, followed by the Midwest and Serra Catarinense regions.

**Conclusions:**

Drug poisoning in children is predominant in early childhood, mainly caused by accidental ingestion of drugs at home. These findings highlight the importance of preventive and educational measures among family members and caregivers.

## RESUMO

**Objetivo:**

Analisar a incidência das intoxicações medicamentosas em crianças registradas no Centro de Informação e Assistência Toxicológica de Santa Catarina entre os anos de 2016 e 2020.

**Métodos:**

Estudo epidemiológico observacional, com delineamento de coorte histórica. Foi realizado com base nos casos notificados de intoxicação por medicamentos em crianças de zero a 12 anos. A amostra foi do tipo censo.

**Resultados:**

Foram notificadas 4.839 intoxicações medicamentosas em crianças no Estado de Santa Catarina no período, com taxa de incidência média anual de 6 casos/mil nascidos vivos. A idade apresentou mediana de três anos. A maioria (51,5%) dos casos de intoxicação ocorreu entre meninas, até os três anos de idade, de causa acidental, por exposição oral e no domicílio. Houve predomínio de sinais e sintomas que afetaram o sistema nervoso e apenas uma pequena parcela (6,2%) necessitou de hospitalização. A maioria dos casos (65,6%) foi considerada intoxicação leve com evolução favorável. Nenhum óbito foi registrado. Houve tendência de aumento dos casos ao longo do tempo, porém não significativo. Observa-se predomínio de casos incidentes no Grande Oeste, seguido do Meio-Oeste e Serra Catarinense.

**Conclusões:**

As intoxicações medicamentosas em crianças predominam na primeira infância, de forma acidental, sendo o ambiente doméstico o principal local. Esses achados destacam a importância de intensificar medidas preventivas e educativas entre familiares e cuidadores de crianças.

## INTRODUCTION

Medicines are vital for the treatment and prevention of diseases because they have prophylactic, curative, palliative, substitutive, or diagnostic purposes.^1^ They must be safe, effective, affordable for the patient, and meet the quality criteria.^2,3^ Both the irrational prescription and the incorrect use of drugs without medical advice are risk factors for human poisoning. Children are very susceptible to this event due to several factors, such as the inability to consider and inhibit risky situations, their neurological immaturity, great curiosity, and tendency to repeat adult behaviors. In addition to their natural active behavior and oral habits in early childhood, they still suffer from the effects of inappropriate use and medical prescription errors.^4^

As pharmacological resources advance to combat diseases, the number of occurrences related to adverse reactions and drug poisoning has also increased.^5^ In recent years, studies have shown that morbidity and mortality related to these occurrences have become a major public health problem, raising discussions about patient safety in taking medicines and the search for satisfactory therapeutic outcomes.^6,7^ Adverse drug reactions are a common cause of demand for emergency services and have a high annual cost.^8^ Drug poisoning is a leading cause of notifications by the Centers for Information and Toxicological Assistance (CIAT) in Brazil.^9^

There is a shortage of pharmacokinetic studies with a pediatric focus, which may cause prescription and dosage errors. In addition, to address this problem, some prescriptions are adapted to allow the use of the drug in children, such as changing doses of drugs designed for adults, opening capsules to mix with food, crushing tablets, and modifying the routes of medication administration. Even when well-executed, such adaptations involve risks of errors and considerable failures in the final use. Furthermore, the risk of adverse effects and dose errors may increase because of the scarcity of information in the literature to support such practices and insufficient information about the stability, compatibility, and bioavailability of these products.^10^

These multifactorial conditions can cause drug poisoning in children, which is one of the most frequent toxicological emergencies. Children, during their development, go through phases of exploration and are attracted by everything that sparks their curiosity, with great potential for domestic accidents. At home, some people have the habit of leaving medicines in unsafe environments. In most cases, medicines have attractive colors, packaging, and formats, thus contributing to poisoning.^11^ These accidents could be prevented through educational interventions and the implementation of preventive programs, thus reducing the incidence and mortality rates caused by these circumstances.^12,13,14,15^

Based on the high prevalence of drug poisoning in children and the avoidable nature of the circumstances, this study aims to analyze the incidence of drug poisoning in children registered at the Santa Catarina Toxicological Information and Assistance Center between 2016 and 2020.

## METHOD

In this observational epidemiological study, with a historical cohort design, the reports of drug poisoning in children aged 0–12 years, registered at the Centro de Informação e Assistência Toxicológica de Santa Catarina (CIATox/SC), between January 1, 2016, and December 31, 2020, were studied. Census sampling was used to collect data. Records that contained more than 30% of missing information in the notification and those among non-residents in the State of Santa Catarina were excluded.

After ethical approval of the study, the anonymized database was received digitally for analysis. Variables of interest included year and month of notification, sex of the child, age in months, city of residence, drug or substance involved, pharmacological class, circumstance of exposure, route of exposure, clinical manifestations (signs and symptoms), place of occurrence of poisoning, whether there was hospitalization, length of stay, final severity classification (based on the Poisoning Severity Score by the World Health Organization, provided in the notification), and case outcome.

Drugs causing poisoning were classified by the Anatomical Therapeutic Chemical (ATC), according to which drugs are divided into different groups based on their sites of action and therapeutic and chemical characteristics. The classification of the main group corresponding to the anatomical group was used. The drug with the highest frequency in the notifications was described in a table.^16^

The SPSS statistical software (version 21.0, SPSS Inc., Chicago, IL, USA) was used for data processing and analysis. The charts were created in Microsoft Office Excel. Data on live births from each of the 295 municipalities of Santa Catarina were used, based on the data from the National System of Live Births in the public domain, in the study period, to calculate the incidence rate by the municipality. To analyze the distribution of the incidence of cases in the State of Santa Catarina, geoprocessing techniques were used, with the aid of the Geographic Information Systems Software QGS.

Descriptive epidemiology was used for data presentation. The differences between means were examined using one-way analysis of variance (ANOVA) and Tukey’s post hoc test. Linear regression analysis and Pearson’s correlation test were used to verify the temporal trend of poisoning cases over the study period. The variables analyzed were mapped thematically, using the representation of stratification by classes, performed by the method of “natural Jenks breaks”. Significance was set at 5%.

This study was submitted and approved by the Research Ethics Committee of the Universidade do Sul de Santa Catarina, CAAE 53878321.4.0000.5369, opinion number 5,172,056, on December 16, 2021.

## RESULTS

During the study period, 5154 cases of drug poisoning in children were reported; however, 315 (6.1%) were excluded because the subjects were not residents of the State of Santa Catarina. Therefore, 4839 cases of children reported with drug poisoning in the State of Santa Catarina were included in this study, which represented an average annual incidence rate of six cases per thousand live births. The median age was 3 years, ranging from 0 to 144 months (0–12 years of age). Table 1 presents the demographic and clinical characteristics of cases of drug poisoning in children, reported in Santa Catarina, between 2016 and 2020.

**Table 1.** Sociodemographic characteristics of drug poisoning in children in Santa Catarina, 2016–2020 (n=4839).

	n	%
Gender		
Female	2480	51.3
Male	2355	48.7
No information	4	0.08
Age (months)		
0–36	3204	66.2
37–72	978	20.2
73–120	347	7.2
121–144	310	6.4
Circumstance*		
Accidental	3462	71.5
Medication error	783	16.2
Violence	225	4.7
Self-medication	129	2.7
Therapeutic use	96	1.9
Adverse reaction	82	1.7
Misuse	37	0.76
Breastfeeding	9	0.19
Other	8	0.17
No information	60	1.2
Exposure route*		
Oral	4614	95.4
Intravenous	86	1.8
Nasal	45	0.93
Cutaneous	27	0.56
Respiratory/inhalation	26	0.54
Ocular	24	0.50
Rectal/vaginal	2	0.04
Placental	2	0.04
Other	4	0.08
No information	24	0.50
Place of occurrence		
Home	4634	95.8
Health service	114	2.4
Public place	18	0.37
School/nursery	13	0.27
Other	13	0.27
No information	47	0.97

Source: CIATox-SC; *Some notifications had more than one response category.

Most cases of poisoning occurred with a higher prevalence among girls than boys up to 3 years of age, due to accidental causes, oral exposure, and occurring at the child’s home. The mean age was 34 months for accidental drug poisoning, 43 months for medication errors (including self-medication, adverse reaction, and incorrect use), and 135 months for cases of violence (including suicide attempt or homicide), with a statistically significant difference between groups (p<0.001). Table 2 presents the clinical aspects and medical assistance in the cases of drug poisoning in children.

**Table 2.** Clinical characteristics and medical care among drug-intoxicated children in Santa Catarina, 2016–2020 (n=4,839).

Clinical manifestations	n	%
Yes	2066	42.69
No	2773	57.31
Sign and symptoms*		
Neurological	1635	33.79
Gastrointestinal	641	13.25
Cardiovascular	544	11.24
Pain	163	3.37
Dermatological	111	2.29
General malaise	102	2.11
Ocular	96	1.98
Muscular	86	1.78
Respiratory	59	1.22
Body temperature change	54	1.12
Sweating	42	0.87
General sweating	39	0.81
Renal	12	0.25
Metabolic	7	0.14
Other	67	1.38
No information	2773	57.31
Hospitalizaton		
Yes	299	6.18
No	4446	91.88
No information	94	1.94
Time of hospitalization (days)		
<1	28	0.58
1–2	140	2.89
3–4	28	0.58
5–6	7	0.14
7–10	6	0.12
>10	3	0.06
No information	181	3.74
Not applicable	4446	91.89
Severity score		
Mild	3174	65.60
Moderate	104	2.15
Major	33	0.68
Null	1404	29.01
No information	124	2.56
Outcome		
Asymptomatic	1398	28.89
Cure	3237	66.89
Differential diagnosis	119	2.46
No information	85	1.76

Source: CIATox-SC. *Some notifications had more than one response category.

There was a predominance of signs and symptoms affecting the nervous system, followed by the gastrointestinal and cardiovascular systems. Few cases required hospitalization, and, in most cases, the duration of hospitalization was between 1 and 2 days. Most cases were considered mild poisoning with a favorable outcome. No deaths were recorded in the studied sample.

In all, 5360 drugs were identified as the causing agent for poisoning, with an average of 1.1 drugs per child. There was a positive correlation between children’s age and the number of medications used (r=0.229; p<0.001). Table 3 presents the classes according to the ATC Classification of drugs related to cases of poisoning in children and their main representative drug. In several cases (n=298), there was a record of the use of more than one drug or pharmacological class involved.

**Table 3.** Drug classes and main representative names.

	ATC classification	n	%	Main medicine name (n)
A	Alimentary tract and metabolism	335	6.3	Bromopride (31)
B	Blood and blood forming organs	73	1.4	Ferrous sulfate (9)
C	Cardiovascular system	249	4.7	Losartan (53)
D	Dermatological	256	4.8	Potassium permanganate (29)
G	Genito urinary system and sex hormones	188	3.5	Levonorgestrel/Ethinylestradiol (97)
H	Systemic hormonal preparations	225	4.2	Sodium levothyroxine (99)
J	Anti-infectives for systemic use	245	4.6	Amoxicillin (108)
L	Antineoplastic and immunomodulation agents	12	0.2	Cyclosporin and methotrexate (6)
M	Musculo-skeletal system	479	8.9	Ibuprofen (277)
N	Nervous system	2275	42.4	Clonazepam (521)
P	Antiparasitic products, insecticides, and repellents	55	1.0	Nitazoxanide (22)
R	Respiratory system	779	14.5	Phenylephrine (142)
S	Sensory organs	30	0.6	Brimonidine (9)
V	Various	159	2.9	Acriflavine; atropa belladonna; methylene blue; methenamine (78)
Total		5360	100.0	

**Figure 1.**
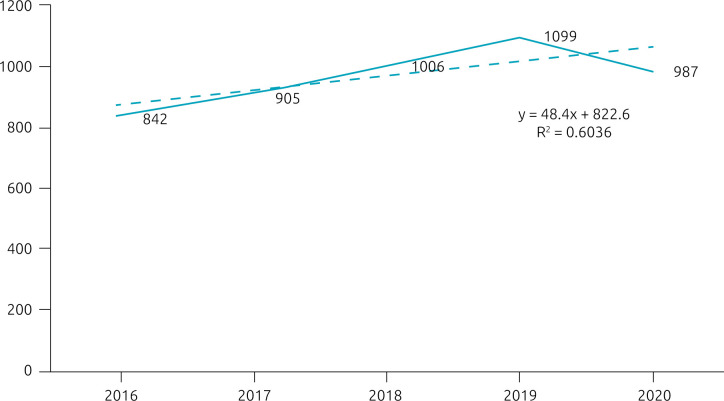
Temporal trend of drug poisoning among children in Santa Catarina, 2016–2020 (n=4,839).

Figure 1 shows the temporal distribution of reported cases of poisoning in children. There was an annual increase in notifications over 4 years under study, with a drop in 2020; however, this increase was not significant (p=0.122). Although the data showed the highest number of cases of drug poisoning during July, August, and October, there was no significant seasonality.

**Figure 2.**
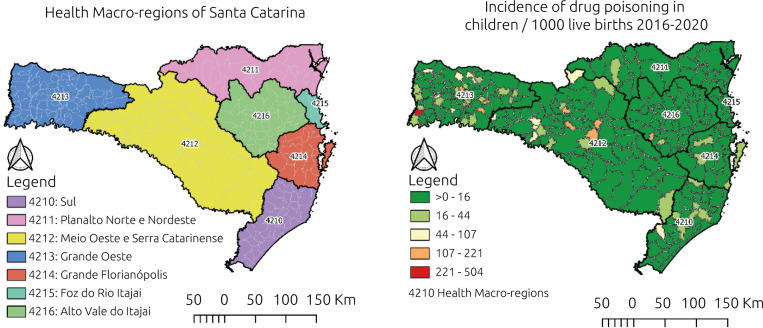
Spatial distribution of reported cases of drug poisoning among children in Santa Catarina, 2016–2020 (n=4,839).

Figure 2 shows the geographical distribution of drug poisoning cases by place of residence of the child. There was a predominance of incident cases in the Great West, followed by the Midwest and Serra Catarinense regions.

## DISCUSSION

Exogenous poisoning is an important public health problem, especially in pediatric age groups, in Brazil and worldwide.^17^ The results of this study highlight the importance of these events and show relevant information about the agents involved. Based on the analyzed data, there was a predominance of notifications for drug poisoning in females, although it was a small difference when compared to males. In addition, a higher occurrence of cases was observed in the 0–6 age group, with a peak in children aged 3 years. It is the fact that children move more easily at this age and are vulnerable to accidents, which may be a rationale for such a result. The surveyed age group corroborates the results obtained in another study carried out in the country,^18^ with a gender-related difference, given that the study by Leite et al. showed a higher occurrence of cases in boys.

Regarding the circumstances, accidental poisoning was the most frequent, which was also found by other authors.^18–20^ These data expose the need for strict precautions to prevent poisoning in children, as they are more likely to have accidental episodes. In addition, poisoning may occur due to different circumstances, among them, medication errors. Approximately 8% of medication errors identified in different national and international databases refer to the pediatric population, mainly in case reports related to overdosing.^21^ Such vulnerability is due to factors such as the difficulty of performing several calculations in various phases of the medication process (prescription, dispensing, preparation, monitoring, and administration) and the use of fractioned doses.^21^ Poisoning via breastfeeding although presenting low rates in this study is something extremely important to be discussed. Medicines can be secreted through the milk and cause problems for the newborn, so discontinuing the use of medicines during gestation and breastfeeding is highly recommended.^22^ The correlation between age and the greater number of medications used is noteworthy, especially related to suicide attempt. Recent studies reveal an increase in cases of mental illness in children and adolescents.^23^

In this study, the oral route of poisoning was the most commonly reported. Similarly to other studies, the home environment was the predominant site of exposure (95.76%), due to inadequate storage, the so-called home pharmacies, which facilitate access for children and adolescents.^18,19^ A study conducted on children and adolescents found that education programs for parents and caregivers to prevent exposure to pharmacological drugs were not successful. Medicines continue to cause harm due to unsafe storage, which facilitates exposure and unintentional ingestion, in age groups under 5 years, and also the intentional ingestion of adolescents owing to misuse and abuse.^24^ This situation supports the findings of the study by Martins et al., which showed that the prevalence of Brazilian households with medicines available to children is extremely high (76%), being much higher than in the United States (30%), Belgium (33%), and Serbia (20%).^25^ However, the health service is also responsible for a high incidence of poisoning in children, with medication errors being the most frequent at the time of medication prescription and are related to their readability and inappropriately prescribed medicines.^5,6^

Among the signs and symptoms, the neurological manifestations were dominant. However, such manifestations depended on the class of drug responsible for the poisoning. As in this study, there was a predominance of the nervous system class of drugs, the preponderance of such a manifestation is justified. In this study, only a small portion of children needed hospital admission, and, when necessary, the length of stay was relatively short, typical of an acute and non-serious situation. Furthermore, mild cases and those that progressed to cure were the majority. Other studies also pointed out that most cases evolved with healing, without sequelae.^26,27^ This circumstance can happen due to the low dose of the drug ingested, agility in medical care, or rapid perception of danger by caregivers.

Considering the main pharmacological classes related to the toxicological accidents observed in this study, a greater participation of the nervous system class was verified, with clonazepam being the most frequent representative of this class. This finding is similar to those of the research carried out in 57 pediatric emergency departments in Spain, in which the groups of central nervous system depressants and clonazepam were the most commonly involved, which are included in the group of benzodiazepine psychotropic drugs.^28^ Although the main class involved was benzodiazepine, it is worth remembering that painkillers and antipyretics are frequently used, being also responsible for these accidents and belonging to this ATC class. Furthermore, medicines for the respiratory system also presented a high frequency of cases, which highlights the need for greater control over prescription, marketing, and adequate packaging in the home environment.

Regarding the distribution of cases in the study period, there was a decrease in cases of poisoning in 2020, although not significant, which may be related to the COVID-19 pandemic, in which families in social isolation may have prevented children’s access to medicines in their homes. However, this hypothesis lacks scientific evidence to be confirmed, and it may also have occurred by chance.

Regarding the distribution of reported cases in the state of Santa Catarina, the Midwest and Serra Catarinense (cities of Frei Rogério, Ibiam, and Brunópolis) and Grande Oeste (cities of Tunápolis, Sul Brasil, and Lageado Grande) had the highest rates of poisoning incidence. One of the hypotheses may be the greater distance and access to health services, in addition to cultural differences, peculiar of the interior of the state in relation to the coastal region, which is more populous and socially developed, with a large concentration in urban areas.^29^

Among the limitations of this study, the use of secondary data stands out, depending on the quality of information obtained, based on attendance records, not being possible to control the primary data, and the gaps found. In addition, the data may be underestimated, as not all cases of poisoning may have been reported. Furthermore, this study is a state census, with a 5-year recall, based on the notifications made to the official government agency that portrays a good part of the assistance provided in cases of drug poisoning, which favors the representativeness of the findings and excellent sample size.

In conclusion, pharmaceutical ingestions are highly prevalent in exogenous poisoning in early childhood, accidentally, and at home. The lack of guidance and awareness by those responsible and the ease of access to medicines make the number of poisoning increase in our country. Therefore, considering the analysis of the cases reported in this study, it is emphasized the intensification of guidance on the correct way of use, storage, and disposal of medicines. In addition, people should be warned against the risks of self-medication and the need to implement ways to prevent toxicological accidents in children. In order to avoid serious outcomes, the importance of reporting cases with quality information is also highlighted.

## Data Availability

The database that originated the article is available with the corresponding author.
